# Supplementary material for: Magnesium Supplementation Attenuates Ultraviolet-B-Induced Damage Mediated through Elevation of Polyamine Production in Human HaCaT Keratinocytes
Source: Cells. 2022 Jul 22;11(15):2268. doi: 10.3390/cells11152268 (PMC9332241; doi:10.3390/cells11152268)
Supplement: Supplementary file 1 [file cells-11-02268-s001.zip › cells-1767844-supplementary.pdf]

Table S1　List of genes with change over 2-fold in DNA microarray analysis

No.	Gene name	Gene symbol	Gene accession	Log2Ratio
1	adenosylmethionine decarboxylase 1	AMD1	NM_001287214	1.035169303
2	ATPase family, AAA domain containing 5	ATAD5	NM_024857	1.04531026
3	breast cancer 2, early onset	BRCA2	NM_000059	1.046406039
4	carbamoyl-phosphate synthetase 2, aspartate transcarbamylase, and dihydroorotase	CAD	NM_001306079	1.120820279
5	cell division cycle 6	CDC6	NM_001254	1.868031759
6	cell division cycle associated 7	CDCA7	NM_031942	1.467525535
7	centromere protein V	CENPV	NM_181716	1.05128641
8	chromatin licensing and DNA replication factor 1	CDT1	NM_030928	1.001201027
9	claspin	CLSPN	NM_001190481	1.274924812
10	clustered mitochondria (cluA/CLU1) homolog	CLUH	NM_015229	1.039414206
11	coiled-coil domain containing 86	CCDC86	NM_024098	1.04213557
12	CTP synthase 1	CTPS1	NM_001301237	1.043056896
13	cysteine and histidine rich domain containing 1 pseudogene	LOC727896	NR_026659	1.052933699
14	denticleless E3 ubiquitin protein ligase homolog (Drosophila)	DTL	NM_001286229	1.524137957
15	DNA replication and sister chromatid cohesion 1	DSCC1	NM_024094	1.244801489
16	ets variant 4	ETV4	NM_001079675	1.034339481
17	family with sequence similarity 111, member B	FAM111B	NM_001142703	1.432668726
18	general transcription factor IIH subunit 2	GTF2H2	NM_001515	1.472671562
19	GINS complex subunit 2 (Psf2 homolog)	GINS2	NM_016095	1.543618693
20	heat shock protein 90kDa alpha (cytosolic), class B member 3, pseudogene	HSP90AB3P	AY956764	1.019893602
21	matrix metallopeptidase 1	MMP1	NM_001145938	1.085427507
22	matrix metallopeptidase 7	MMP7	NM_002423	1.379956995
23	microRNA 1244-1	MIR1244-1	NR_036052	1.073481518
24	minichromosome maintenance complex component 6	MCM6	NM_005915	1.180015648
25	minichromosome maintenance 10 replication initiation factor	MCM10	NM_018518	1.212731378
26	neuropilin (NRP) and tolloid (TLL)-like 2	NETO2	NM_001201477	1.069822455
27	origin recognition complex subunit 1	ORC1	NM_001190818	1.083430959
28	parathyroid hormone-like hormone	PTHLH	NM_002820	1.265984421
29	polymerase (DNA directed), alpha 2, accessory subunit	POLA2	NM_002689	1.046318516
30	polymerase (RNA) III (DNA directed) polypeptide G (32kD)	POLR3G	NM_006467	1.388402734
31	pseudouridylate synthase 7 (putative)	PUS7	NM_019042	1.227101246
32	pumilio RNA binding family member 3	PUM3	NM_014878	1.074525598
33	serpin peptidase inhibitor, clade B (ovalbumin), member 3	SERPINB3	NM_006919	1.632277396
34	serpin peptidase inhibitor, clade B (ovalbumin), member 4	SERPINB4	NM_002974	1.618956264
35	small ILF3/NF90-associated RNA D	SNAR-D	NR_024243	1.087644061
36	small ILF3/NF90-associated RNA E	SNAR-E	NR_024258	1.097734351
37	small ILF3/NF90-associated RNA H	SNAR-H	NR_024342	1.024805471
38	small nucleolar RNA, H/ACA box 50C	SNORA50C	NR_002995	1.313952675
39	solute carrier family 43, member 3	SLC43A3	NM_001278201	1.13375756
40	spermidine synthase	SRM	NM_003132	1.024913924
41	TIMELESS interacting protein	TIPIN	NM_001289986	1.09687562
42	translocase of outer mitochondrial membrane 40 homolog (yeast)	TOMM40	NM_001128916	1.067885485
43	uncharacterized LOC101927746	LOC101927746	NR_110115	1.004901758
44	VPS9D1 antisense RNA 1	VPS9D1-AS1	NR_036480	1.206086224
45	zinc finger protein 92	ZNF92	NM_001287532	1.065628666

Table S2 List of genes with change less than half in DNA microarray analysis

No.	Gene name	Gene accession	Gene symbol	Log2Ratio
1	acid phosphatase, prostate	NM_001099	ACPP	-1.005841376
2	cadherin 5, type 2 (vascular endothelium)	NM_001795	CDH5	-1.019344087
3	cyclin-dependent kinase inhibitor 2B (p15, inhibits CDK4)	NM_004936	CDKN2B	-1.155954011
4	keratin associated protein 20-1	NM_181615	KRTAP20-1	-1.178930232
5	microRNA 4454	NR_039659	MIR4454	-1.121444013
6	phosphoinositide-3-kinase interacting protein 1	NM_001135911	PIK3IP1	-1.108284718
7	small nucleolar RNA, H/ACA box 38B	NR_003706	SNORA38B	-1.009536958
8	small nucleolar RNA, H/ACA box 80A	NR_002996	SNORA80A	-1.199736683
9	solute carrier family 30 (zinc transporter), member 1	NM_021194	SLC30A1	-1.097828811
10	thioredoxin interacting protein	NM_001313972	TXNIP	-1.212522437
11	uncharacterized LOC100134868	NR_004846	LOC100134868	-1.382168943
12	uncharacterized LOC101928152	XR_245037	LOC101928152	-1.017932323
13	uncharacterized LOC105377957	XR_942895	LOC105377957	-1.100078201

Table S3 Gene ontology analysis of differentially expressed genes in high $MgCl_2$-treated cells

Expression	Category	Term	Count	%	PValue	FDR
Upregulated	GOTERM_BP_DIRECT	GO:0006260~DNA replication	7	15.6	9.07E-08	2.10E-05
	GOTERM_BP_DIRECT	GO:0006270~DNA replication initiation	5	11.1	2.04E-07	2.37E-05
	GOTERM_BP_DIRECT	GO:0000076~DNA replication checkpoint	4	8.9	7.14E-07	5.52E-05
	GOTERM_BP_DIRECT	GO:0033314~mitotic DNA replication checkpoint	3	6.7	1.51E-04	0.008786389
	GOTERM_BP_DIRECT	GO:0009411~response to UV	3	6.7	0.00319852	0.148411311
	GOTERM_BP_DIRECT	GO:0051301~cell division	5	11.1	0.005440387	0.185548714
	GOTERM_BP_DIRECT	GO:0008295~spermidine biosynthetic process	2	4.4	0.005598453	0.185548714
	GOTERM_BP_DIRECT	GO:0033262~regulation of nuclear cell cycle DNA replication	2	4.4	0.009313813	0.240089396
	GOTERM_BP_DIRECT	GO:0006595~polyamine metabolic process	2	4.4	0.009313813	0.240089396
	GOTERM_BP_DIRECT	GO:0006974~cellular response to DNA damage stimulus	4	8.9	0.014673761	0.340431244
	GOTERM_BP_DIRECT	GO:0048478~replication fork protection	2	4.4	0.016704089	0.352304414
	GOTERM_BP_DIRECT	GO:0000727~double-strand break repair via break-induced replication	2	4.4	0.022211594	0.400069917
	GOTERM_BP_DIRECT	GO:1900264~positive regulation of DNA-directed DNA polymerase activity	2	4.4	0.024040755	0.400069917
	GOTERM_BP_DIRECT	GO:0010466~negative regulation of peptidase activity	2	4.4	0.025866589	0.400069917
	GOTERM_BP_DIRECT	GO:0030174~regulation of DNA-dependent DNA replication initiation	2	4.4	0.025866589	0.400069917
	GOTERM_BP_DIRECT	GO:0007049~cell cycle	4	8.9	0.029144963	0.422601965
	GOTERM_BP_DIRECT	GO:0033044~regulation of chromosome organization	2	4.4	0.034946063	0.476910976
	GOTERM_BP_DIRECT	GO:0006541~glutamine metabolic process	2	4.4	0.040354198	0.514677998
	GOTERM_BP_DIRECT	GO:0006268~DNA unwinding involved in DNA replication	2	4.4	0.042150353	0.514677998
	GOTERM_BP_DIRECT	GO:0008283~cell proliferation	3	6.7	0.047179278	0.547279622
	GOTERM_CC_DIRECT	GO:0005654~nucleoplasm	21	46.7	4.23E-06	2.96E-04
	GOTERM_CC_DIRECT	GO:0005634~nucleus	25	55.6	1.24E-05	4.32E-04
	GOTERM_CC_DIRECT	GO:0005694~chromosome	6	13.3	1.05E-04	0.002457948
	GOTERM_CC_DIRECT	GO:0031298~replication fork protection complex	2	4.4	0.005558523	0.097274144
	GOTERM_CC_DIRECT	GO:0071162~CMG complex	2	4.4	0.02023458	0.283284121
	GOTERM_CC_DIRECT	GO:0000781~chromosome, telomeric region	3	6.7	0.040921179	0.454118046
	GOTERM_CC_DIRECT	GO:0051233~spindle midzone	2	4.4	0.045411805	0.454118046
	GOTERM_MF_DIRECT	GO:0003688~DNA replication origin binding	4	8.9	1.11E-05	8.33E-04
	GOTERM_MF_DIRECT	GO:0003677~DNA binding	9	20.0	0.002521601	0.094560055
	GOTERM_MF_DIRECT	GO:0019899~enzyme binding	5	11.1	0.006346827	0.15867067
	GOTERM_MF_DIRECT	GO:0002020~protease binding	3	6.7	0.018437085	0.305796999
	GOTERM_MF_DIRECT	GO:0003697~single-stranded DNA binding	3	6.7	0.020386467	0.305796999
	GOTERM_MF_DIRECT	GO:0016887~ATPase activity	4	8.9	0.029204609	0.365057616
	GOTERM_MF_DIRECT	GO:0017116~single-stranded DNA-dependent ATP-dependent DNA helicase activity	2	4.4	0.039449939	0.422677914
Downregulated	GOTERM_BP_DIRECT	GO:0006874~cellular calcium ion homeostasis	2	18.2	0.039314757	1
	GOTERM_CC_DIRECT	GO:0031965~nuclear membrane	2	18.2	0.090401909	1

Table S4 KEGG pathway analysis of differentially expressed genes in high $MgCl_2$-treated cells

Pathway ID	Term	Count	%	PValue	Genes
hsa04110	Cell cycle	3	6.7	0.027675685	NM_005915, NM_001254, NM_001190818
hsa03030	DNA replication	2	4.4	0.072513373	NM_005915, NM_002689
hsa00330	Arginine and proline metabolism	2	4.4	0.099352663	NM_003132, NM_001287214

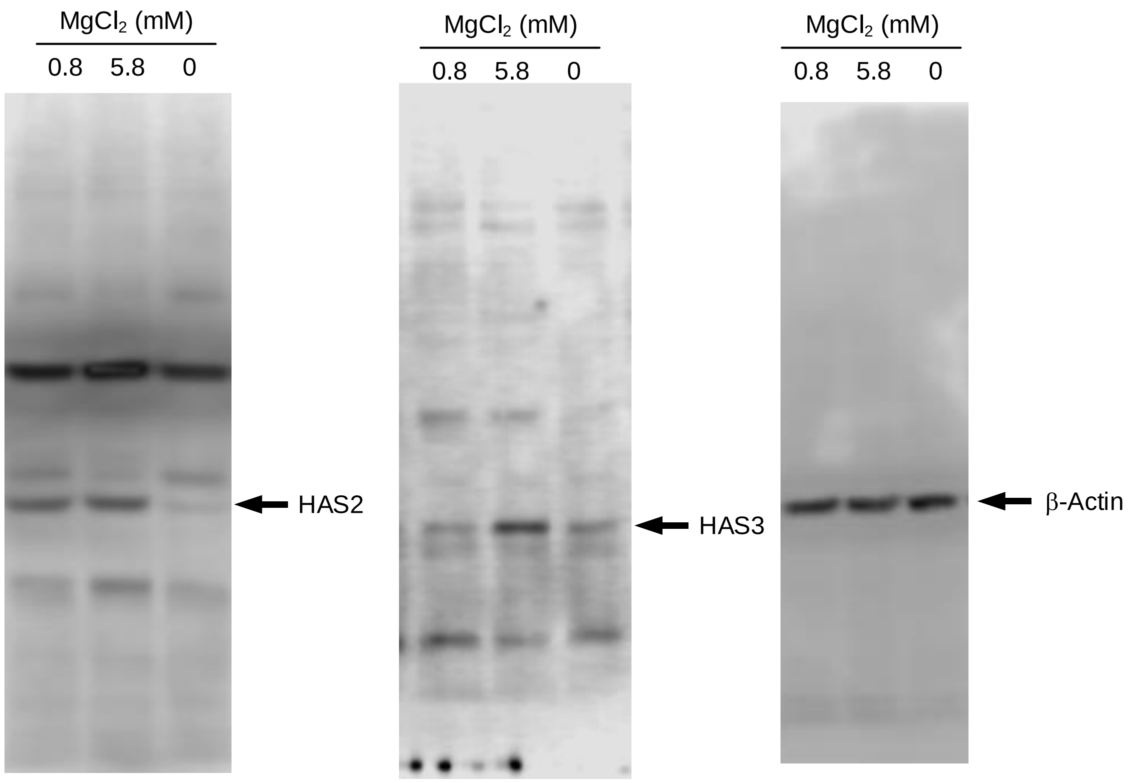

Figure S1 Original Images of figure 1C

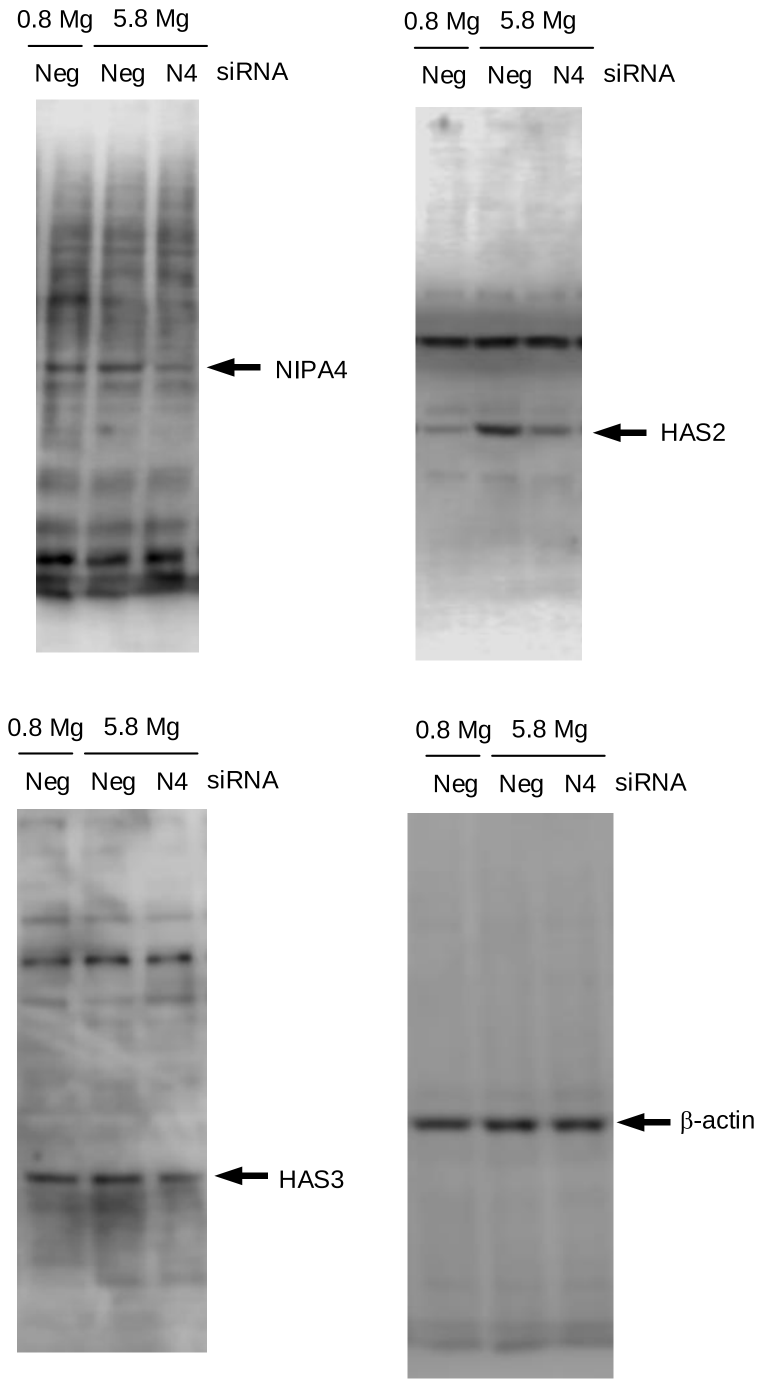

Figure S2　Original Images of figure 2C

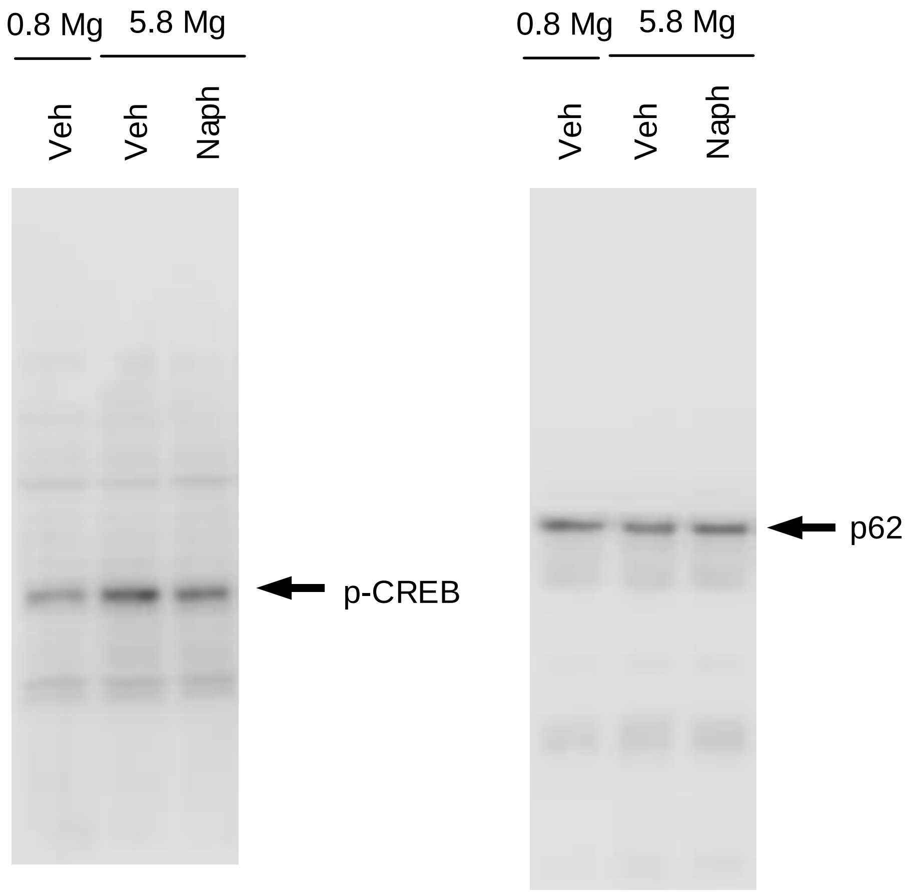

Figure S3　Original Images of figure 8A
